# Dietary Intervention by Phytochemicals and Their Role in Modulating Coding and Non-Coding Genes in Cancer

**DOI:** 10.3390/ijms18061178

**Published:** 2017-06-01

**Authors:** Liviuta Budisan, Diana Gulei, Oana Mihaela Zanoaga, Alexandra Iulia Irimie, Sergiu Chira, Cornelia Braicu, Claudia Diana Gherman, Ioana Berindan-Neagoe

**Affiliations:** 1 Research Center for Functional Genomics, Biomedicine and Translational Medicine, University of Medicine and Pharmacy “Iuliu-Hatieganu”, 400012 Cluj-Napoca, Romania; lbudisan@yahoo.com (L.B.); oana.zanoaga@umfcluj.ro (O.M.Z.); sergiu.chira@umfcluj.ro (S.C.); cornelia.braicu@umfcluj.ro (C.B.); ioana.neagoe@umfcluj.ro (I.B.N.); 2 MEDFUTURE-Research Center for Advanced Medicine, University of Medicine and Pharmacy “Iuliu-Hatieganu”, 400012 Cluj-Napoca, Romania; gulei.diana@umfcluj.ro; 3 Department of Prosthodontics and Dental Materials, Faculty of Dental Medicine, University of Medicine and Pharmacy “Iuliu Hatieganu”, 23 Marinescu Street, 400012 Cluj-Napoca, Romania; irimie.alexandra@umfcluj.ro; 4 Surgical Clinic II, 4–6 Clinicilor Street, 400006 Cluj-Napoca, Romania; 5 Department of Surgery, University of Medicine and Pharmacy “Iuliu Haţieganu”, 8 Victor Babes Street, 400012 Cluj-Napoca, Romania; 6 Department of Functional Genomics and Experimental Pathology, Oncological Institute “Prof. Dr. Ion Chiricuţă”, 400015 Cluj-Napoca, Romania

**Keywords:** phytochemicals, cancer, apoptosis, coding and non-coding RNA, miRNAs

## Abstract

Phytochemicals are natural compounds synthesized as secondary metabolites in plants, representing an important source of molecules with a wide range of therapeutic applications. These natural agents are important regulators of key pathological processes/conditions, including cancer, as they are able to modulate the expression of coding and non-coding transcripts with an oncogenic or tumour suppressor role. These natural agents are currently exploited for the development of therapeutic strategies alone or in tandem with conventional treatments for cancer. The aim of this paper is to review the recent studies regarding the role of these natural phytochemicals in different processes related to cancer inhibition, including apoptosis activation, angiogenesis and metastasis suppression. From the large palette of phytochemicals we selected epigallocatechin gallate (EGCG), caffeic acid phenethyl ester (CAPE), genistein, morin and kaempferol, due to their increased activity in modulating multiple coding and non-coding genes, targeting the main hallmarks of cancer.

## 1. Introduction

**Figure 1:**
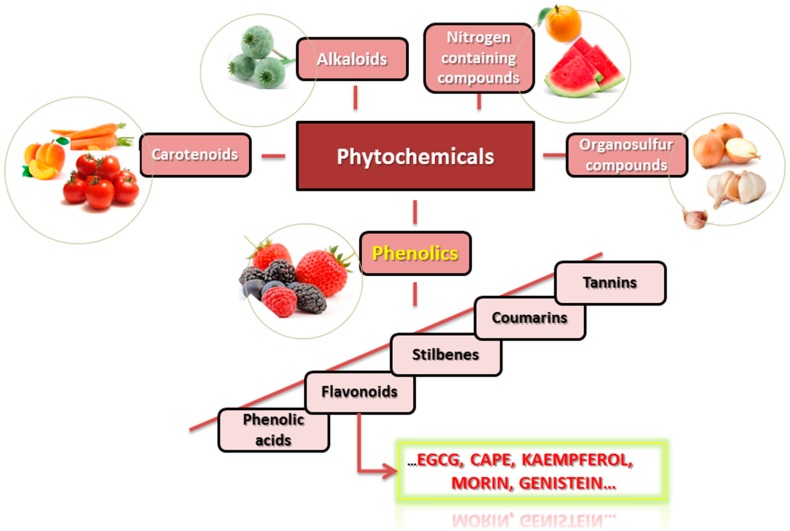
The main classes of phytochemicals and their bioavailability.

Phytochemicals are listed as secondary metabolites that are naturally found in plants with roles involved in the restoration of damaged cells, but also in determination of colour, aroma and taste of the plants. These types of products are classified based on the starting point of their biosynthesis: phenolic compounds, carotenoids, products with nitrogen, alkaloids and organosulfur compounds (Figure 1) [1,2,3,4]. Every class of phytochemicals is further divided in many other smaller subclasses forming a complex diagram of classification, with a wide range of isomeric form and different substituents, that show the different biological active effect [5].

Initially associated with antioxidant properties and prevention of free radicals generation, recent studies reveal a more complex protective action at cellular and molecular levels for natural compounds, with important application in disease prevention or treatment. These evidences are supported by epidemiological studies [5,6,7]. The benefits of natural products are also underlined by the so-called “Asian paradox”. In the attempt to find an eligible reason for the reduced rates of lung cancer within the Asian population, researchers concluded that the high amount of green tea could be the reason. The catechins within the composition of green tea seem to reduce the risk for pulmonary diseases among others, despite the fact that the target population is also included in the list of active smokers. A similar situation is encountered in the case of “French paradox” due to the high intake of products containing resveratrol [6]. This population is characterized by a reduced risk of cardiovascular diseases and the current studies attribute this paradigm to the positive action of the stilbenoid. These population-based studies are contributing to the value of polyphenols as a potent source for new drug discovery, based on their capacity to modulate numerous pathological processes including malignant transformation and development, potentiating their secondary usage for the treatment of chronic diseases, and other health problems [6,7].

A rediscovery of the natural phytochemicals in context of pathological conditions was observed in the past years. This re-emergence is demonstrated by the increased number of publications focused on a better comprehension of their biological function and the complex beneficial properties in human health. This was assessed at a cellular, molecular or genomic level using a wide range of cell lines or animal models. Based on this, phytochemicals were proved to be involved in a wide range of key mechanisms in chemoprevention or chemotherapy. Unfortunately, only a limited number of studies registered a success at the level of clinical trials but with deceptive results [8], due to their low stability. A possible alternative could be their modification in more stable pro-drugs [9].

Natural phytochemicals were used in cancer prevention and therapy in traditional medicine due to their safety, lack of side effects and their bioavailability, from a wide range of natural sources. After the development of last generation research techniques, these natural products were re-evaluated in terms of beneficial effects. Polyphenols were demonstrated to have impact on human health, having the capacity to modulate gene expression, non-coding RNAs (ncRNAs) or epigenetic processes [6], this being backed by the latest progress related to the “omics” approaches [10]. This paper focuses on the emphasis of the role of these classes of phytochemicals in cancer therapy via coding and non-coding related pathways.

## 2. Phytochemical Compounds Activities Are Determined by Their Structure

The multiple experimental studies have revealed the structure-related function of these compounds. The knowledge of the structure-activity relationship has a relevant significance, particularly in the case of developing novel therapeutics derived from natural compounds with application in cancer therapy [6,11]. Knowing the exact purpose of the functional groups could improve the treatment options in accordance with their specific effects at molecular level for a wide range of pathologies, including cancer [11]. In the light of the structure related functions, new computational approaches, like molecular docking assays, are now at the centre of interest for in silico determination of targeted compounds towards cancer inhibition [12].

The chemical structure of polyphenols is characterized by the presence of more than one phenolic group (a hydroxyl group bound to an aromatic ring) per molecule, a structure that provides the antioxidant function [13]. The antioxidant property via hydroxyl groups is able to eliminate the harmful free radicals within the cell, in order to maintain the appropriate physiological status. The function of the additional hydroxyl groups consists of the release of hydrogen molecules that inactivates the free radicals. Therefore, a compound with an increased number of hydroxyl groups has a more accentuated protective activity than a compound with a far less functional groups.

Studies regarding the structure-activity relationship in the case of catechins have shown that the gallate groups, particularly the galloyl groups (at C-3 position), play a major role in their biological activity due specific binding with bovine serum albumin [14,15,16]. The epigallocatechin gallate (EGCG) chemical structure is composed of three aromatic rings, two of them situated in a parallel pattern and the third one perpendicular on the reminded two rings and has a therapeutic effect by inhibition of carbonyl-amine crosslinking reactions [17,18].

Catechnis are metabolised to unstable quinone metabolites, displaying an mechanism of oxidative coupling of the galloyl group with the B-ring leading to quinone dimerization. Similar processes are retrieved in plants responsible for the production of theasinensins [19]. Catechins, containing a pyrogallol moiety, were able to target electrophile-responsive element (EpRE) being related to their metabolisation in quinones targeting important genes involved in the detoxification [20].

For the case of caffeic acid phenethyl ester (CAPE), it was also shown that the specific structure is composed of two phenol hydroxyls groups with a key role in the biologically active process. Moreover, CAPE activity can be increased by substitution of one hydroxyl group and by extension of the alkyl chain of the alcohol part [21]. CAPE has a higher hydrophobicity and powerful inhibition capacity of xanthine oxidase (XO). The inhibition of the enzymatic activity is caused by binding to the molybdopterin region of the active site [22].

One of the most important biological properties of genistein is related to its chemical structure and estrogenic activity, having a free hydroxyl group at position 4′ and 7′ [23]. Antiproliferative and cytotoxic effects are due to inhibition of cellular enzymes through intramolecular hydrogen bonding which make genistein more hydrophobic [23]. The bioactivity of morin is associated with the location of the 5-OH and 4-CO, also with 3-OH and 4-CO groups in a molecule [24]. Structure-function studies have shown that tumour selectivity of morin involves the 2′, 4′ hydroxyl configuration in the B ring [25]. The protective effect of kaempferol is closely linked to the o-dihydroxy structure belonging from B-ring [26]. There is an important role for the antioxidant activity of kaempferol to play when it is combined with the presence of hydroxyl groups at C3, C5 and C4’ [27].

## 3. Antioxidant Benefits of Phytochemicals

Numerous studies have shown the important role of phytochemicals in prevention or treatment for a wide range of diseases (cancer, neurodegenerative diseases, metabolic disease and immune pathologies). The benefits of phytochemicals or nutraceutical compounds from fruit and vegetables are even more powerful than is currently understood and this can be demonstrated. The major role of the phytochemicals consists of antioxidant protection, since in many processes involved in metabolism, a production of reactive oxygen species may result [28] in preventing the DNA, lipids, and proteins damage [29]. In addition, a wide range of phytochemicals were proved to have an important role in regulation of cell proliferation, cell cycle, immune response, or the reducing of lipid oxidation [30,31].

The antioxidant activity was related to the number of hydroxyl substituents retrieved in the B-ring for anthocyanidins; the opposite was observed for catechins. EGCG is represented by the most bioactive flavone-3-ol phenolic compound, this can be attributed to their eight free hydroxyl groups, responsible for the high versatile biological role [32]. The substitution with a methoxyl leads to a reduced antioxidant effect in the case of anthocyanidins compounds. From the main catechins, different isomers types like the *cis*-*trans* isomerism, epimerization, and racemization did not significantly modify the total antioxidant activity [33].

The cytotoxic effects of EGCG were demonstrated to be connected to its auto-oxidation, leading to the generation of hydrogen peroxide or other EGCG auto-oxidation products [34]. The mechanism of auto-oxidation was related to the activation or the mitoagen activated protein kinases (MAPK), particularly the phosphorylation of the ERK1/2 in Jurkat cells [35]. This mechanism can be related to contradictory data available on this compound.

The EpRE mediate the expression of two major defence enzymes NAD(P)H-quinone oxidoreductase (NQO1) and glutathione *S*-transferases (GSTs), that protect against environmental toxic agents that generate reactive oxygen species (ROS) [20]. Catechins have the capacity to generate oxidative stress, and the modulation of intracellular glutathione (GSH) via EpRE activation. Even the pro-oxidant effect caused by catechins can be considered a health promoting effect due to their capacity to activate detoxifying enzymes, via quinone formation [20,36].

## 4. Phytochemical Compounds and Cancer

Phytochemicals modulate coding and non-coding RNA gene expression leading to the restoration of the normal signal transduction pathways [37,38,39]. Phytochemical intervention in chemotherapy are sustained by a higher number of clinical trials that have shown that these compounds increase the treatment efficiency and decrease the side effects, inducing apoptosis in cancer cells, reducing drug resistance and also the severity of comorbid conditions. Some relevant examples are presented in Table 1.

**Figure 2:**
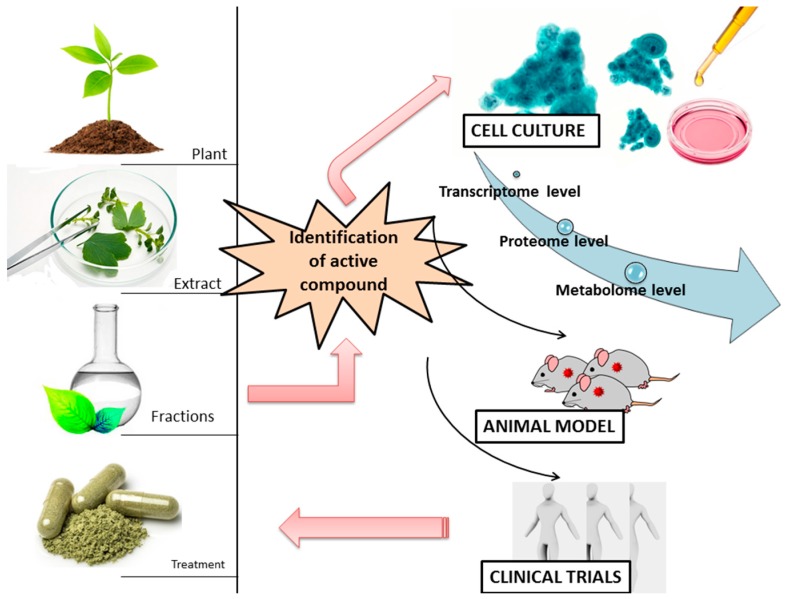
The summary of the workflow in the identification of the novel bioactive agent is extraction, fractionation, then cell culture based test to evaluate the effects at cellular and molecular level of the bioactive extract and validation on animal models of the most relevant finding and the final step of a novel treatment is the clinical trials evaluation.

These compounds can act as pro or anti-oxidant, based on the dose and exposure time as presented by most of the studies [40,41], because they interfere with key cellular processes (cell cycle regulation, apoptosis, or even angiogenesis, invasion and metastatic processes). The phytochemicals-related mechanisms of action are summarized in a simplistic form in Figure 2.

### 4.1. (−)-Epigallocatechin-3-Gallate (EGCG)

(−)-Epigallocatechin-3-gallate (EGCG) is the major component and the most bioactive phenolic constituent of green tea. Based on preclinical evidence, it has multiple biological functions, such as inducing cell apoptosis, inhibiting angiogenesis and suppressing metastasis. EGCG anticancer activity is proved in cancer cell lines and animal tumour models [42,43], but is also found in an increased number of clinical trials that involves their chemo-protective or chemotherapeutic role. In addition to the antioxidant activity, EGCG also acts as a pro-oxidant because of the hydrogen peroxide formation, the dual role of EGCG being dose-related. It is important to mention that tumour cells are more vulnerable to oxidative stress than normal cells; meaning a high specificity of action targets only the altered mechanism on tumoural cells and no cytotoxic effects on normal cells [7]. It was shown that EGCG inhibits the cell growth, migration and invasion in Hs578T triple negative breast cancer cells by repressing the expression of VEGF (vascular endothelial growth factor) pro-angiogenic factor [44,45]. In oral cancers, EGCG compound has a therapeutic role by induction of apoptosis or autophagy [46], limiting cancer cells proliferation [45,47], reduction of cell migration and invasion [48] and modulation of essential transcription factors involved in carcinogenesis [49]. Also in oral cancer cells, the same compound has a general inhibitory role regarding cell proliferation, and this action was preserved over all stages of carcinogenesis [50]. EGCG is able to inhibit the proliferation of immortalized Human Papilloma Virus (HPV) by acting on the G0/G1 phase and stopping the cell cycle [51]. EGCG specifically targets RasGTPase-activating protein-binding protein 1(G3BP1) having chemopreventive effects in lung cancer [52]. In colorectal cancer, EGCG induces the inhibition of cell proliferation via inhibiting the expression of transcription factor HES1 and neurogenic locus notch homolog protein 2 (Notch2) [53]. EGCG inhibits human prostate cancer cell (PC-3) proliferation by PI3-K-dependent signalling pathway [54]. In colon cancer EGCG induces the activation of mitogen-activated protein kinase (MAPK) [55] and Akt pathways [56]. In silico modelling approaches reveals that EGCG physically interacts with the ligand-binding domain of androgen receptor that is overexpressed in prostate cancer, leading to the inhibition of cell growth [57]. These antitumoural mechanisms were related to the modulating acetylation of androgen receptor by anti-histone acetyltransferase activity [58].

### 4.2. Morin (3,2′,4′,5,7-Pentahydroxyflavone)

Morin (3,2′,4′,5,7-pentahydroxyflavone) is a flavone that belongs to the Moraceae plants family and is recognized for its anti-carcinogenic and anti-inflammatory roles in different pathologies, including cancer [25,59,60]. Furthermore, it has been demonstrated to act as a chemopreventive agent in oral malignancies [25,59]. Morin is also involved in inhibition of hepatocytes transformation by suppressing AP-1 activity and inducing S-phase arrest [61]. Apoptosis induced by morin in human leukemia HL-60 cells can involve a mitochondria-dependent pathway and a caspase-3-mediated mechanism [62]. In the MCF-7 line of breast cancer cells, this pentahydroxyflavone can target cell proliferation via caspase-activated mitochondrial pathway or through independent pathways [63]. In a human leukemic cell line, morin treatment was observed to activate the caspase-dependent apoptosis in a dose-dependent mode. At the same time, it was observed to have an effect on mitochondrial membrane potential, connected with the realising of citocrome c, inhibition the expression of Bcl-2 and activation of Bax proteins [64]. Morin is an important apoptotic modulator. In colorectal cancer cells, it was demonstrated that Morin has the capacity to regulate apoptosis via a caspase-dependent mechanism, by upregulating the Fas receptor but also the intrinsic apoptotic pathways via Bcl-2 and cIAP-1, anti-apoptotic proteins. Also, it was able to be involved in ROS generation, which targets the *Akt* gene [65].

Important pro-apoptotic activity was demonstrated in the case of hepatocellular cancer mouse models, chemically induced by diethylnitrosamine (DEN). Therefore, the morin treatment was related with the upregulation of *PTEN* gene, one important gene with tumour suppressor role, recognised as negative regulator of *Akt* [65], also was demonstrated to modulated Bcl-2/Bax ratio, leading to the activation of cytochrome c and overexpression of caspases 3 and 9 [65].

MDA-MB-231, even at low doses, was observed to have the capacity to inhibit colony formation, and was proved to increase the expression level for MMP-9 (Matrix metalloproteinase 9), in parallel with the overexpression of N-cadherin an important epithelial marker and inhibit the activation of Akt pathways. In mouse models, it was observed to have the capacity to reduce cell progression and the antimetastatic effect [66]. In a similar study, it was proved to have EMT features via inhibition VCAM1 and *N*-cadherin expression level. Likewise, in mouse models, it was demonstrated to specifically inhibit lung cancer metastasis [67].

### 4.3. Caffeic Acid Phenethyl Ester (CAPE)

Caffeic acid phenethyl ester (CAPE) is one of the most important bioactive agents retrieved in high concentration in propolis. CAPE has multiple biologic active properties among which we can list: antiviral, antibacterial, antioxidant, anti-inflammatory and overall anti-cancer activities [68,69,70]. CAPE is targeting the NFκB transcription factor, promoting apoptosis in a wide range of cell lines [71,72,73,74]. For example, HL-60 cells of human leukemia subjected to the CAPE treatment revealed increased apoptosis by activation of different regulatory elements caspase-3 and BAX regulator and suppression Bcl-2 [69]. The same inhibitory activity was observed in colon cancer cells (HCT116 and SW480) [75]. Also, in a dose-dependent manner, CAPE decreased the malignant potential of MDA-MB-231 and Hs578T [76], two relevant in vitro models for triple negative breast cancer [70]. Growth inhibition caused by CAPE treatment in PC-3 cells are accompanied by p21Cip1 induction and suppression of Akt signalling [77]. CAPE was demonstrated to act as a biomarker with active role in chemoprevention and chemotherapy in oral cancer patients inhibiting Akt signalling, cell cycle regulatory proteins and NFκB function [78,79]. It has been demonstrated that CAPE has potential application in cervical cancer [80] or ovarian cancer also [74].

### 4.4. Kaempferol

Kaempferol is a natural flavonol, a type of flavonoid found in a variety of plants and plant-derived foods with antioxidant properties. Multiple investigations confirmed the chemo protective effect of these compounds [81,82,83,84]. Thereby, kaempferol promotes apoptosis of ovarian cancer cells through down regulation of cMyc [85]. This natural compound also inhibited pancreatic [86], oral [87], hepatic [88] and breast cancer cell proliferation [84,89,90] by activating different pathways of the apoptosis. In human glioma cells, kaempferol treatment induced inhibition of cell proliferation through caspase-dependent mechanisms [91]. Cell death in leukemia cells as result of kaempferol treatment is accompanied by decreasing the expression of Bcl-2 and increasing the expressions of Bax [92]. An increase in reactive oxygen species (ROS) generation by kaempferol was associated with induction of cell death in human glioma cells [91]. For bladder cancer, kaempferol was demonstrated to have a therapeutic role intermediated by induced expression of PTEN and Akt inhibition [93], or via c-Met/p38 signalling pathway [94], finalized with decreased cell proliferation and accentuated apoptosis [94].

Epithelial-mesenchymal transition (EMT) and metastatic-related comportments of MCF-7 were evidenced to be regulated by kaempferol [95]. These effects were shown to be modulated via regulation of the estrogenreceptos expression [95]. Kaempferol was demonstrated to have the capacity to inhibit TGF-β1-induced EMT and migration by inhibiting Akt1-mediated phosphorylation of Smad3 in lung cancer models [96].

### 4.5. Genistein

Genistein, the major compound of soy, is an isoflavone with bioactive roles and has been revealed to exercise tumour suppressing roles in numerous cancer inhibition mechanisms (apoptosis, cell proliferation, immune response or angiogenesis and invasion) in colon, breast, prostate, lung cancers or hematological malignancies [97,98]. Some of the intermediary signalling pathways targeted by genistein responsible for modulating the anti-tumour activities are: Akt, NFkB, Wnt and p53 [98,99]. In AML cell lines genistein determined increased apoptosis of the cell [100,101] and in CRC lines are suppressing the migratory characteristics of tumour cells [102]. Also this isoflavone acts as tumour suppressor in human breast [103,104] and prostate [99,105] cancer. Also, we have to take into consideration that in some case genistein is related to an increase of the proliferation rate at higher doses [106].

Genistein activates the mitochondrial apoptotic pathways in colorectal cancer cells, by preventing the phosphorylation of Akt protein [107]. In the MCF-7 cells the inhibition of IGF-1R and p-Akt was observed and a downregulation of the Bcl-2/Bax protein ratio as exposure to genistein [108]. A microarray study on breast cancer at different doses revealed different altered pathways and by overlapping the gene networks, the most significant functions were those related to cell cycle [109].

In colorectal cancer cells, genistein was proved to reduce cell proliferation, invasion and metastasis, via downregulated matrix metalloproteinase 2 and FMS-related tyrosine kinase 4, a receptor for the VEGF. The validation on orthotopic mice models, of colorectal tumours, the genistein treatment (oral delivery) did not significantly reduced tumour growth, but was able to prevent distant metastasis [110]. In oral cancer the antimetastatic effect was related by regulating the MMP-2 expression by inhibition of ERK1/2 and the activator protein-1 signalling pathways [111]. At physiological concentration, it was proved to target hormonal receptors [112]. A complex proteomic study reveals that the genistein treatment was related with the alteration of 332 regulated phosphorylation sites on 226 proteins, which are involved in key cellular processes (DNA replication, cohesin complex cleavage, and kinetochore formation) [112].

## 5. Dietary Phytochemicals as Non-Coding Genes Regulators

The term non-coding RNA (ncRNA) is frequently used for those RNA trancripts that do not encode a protein, but are able to regulate gene expression, with important implication in pathological status, including cancer [10,118]. MicroRNAs (miRNAs) are endogenous, small non-coding RNA molecules of 19–25 nucleotide length able to regulate the levels of expression of different genes at the posttranscriptional level [119,120]. Alterations of miRNA expression were observed in many human diseases, especially in cancer where they act as tumour suppressors or oncogenes [118,121,122]. Therefore, manipulation of miRNAs as molecular targets in cancer treatment represents an encouraging method [123]. miRNAs are key transcripts involved in the regulation of transcriptomic and epigenetic mechanisms [123,124]. The effects of natural phytochemicals on modulation of miRNA expression and its related target genes level on different solid tumours are presented in Table 2.

**Figure 3:**
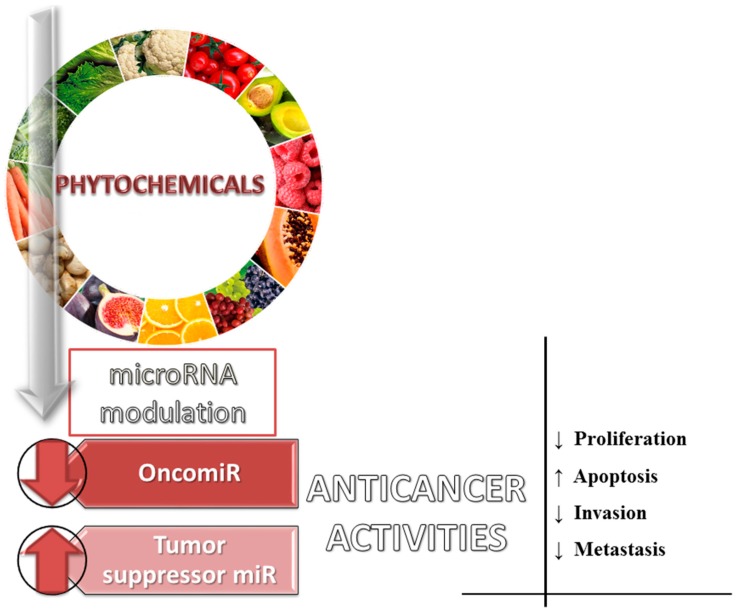
Involvement of dietary phytochemicals in the modulation of oncogenic/tumour suppressor miRNAs, interfering with key cellular and molecular processes (↑: upregulated miRNA; ↓: downregulated miRNAs).

**Figure 4:**
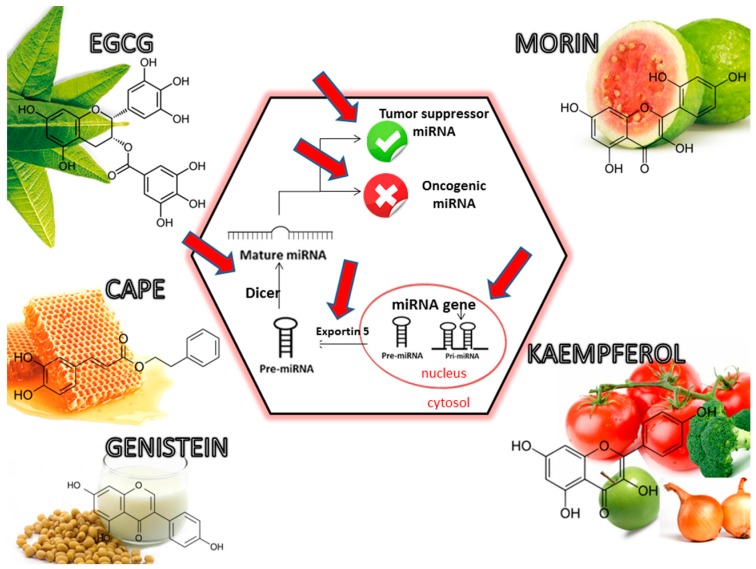
Effects of phytochemicals on regulation the expression of tumour suppressor miRNA and oncomiRNA, with important significance in tumoural pathology. Red arrows display multiple interventional targets of selected phytochemicals on miRNA biogenesis, with important role in the modulation of physiological and pathological processes.

Dietary phytochemicals are recognized to activate or suppress different miRNAs in order to counteract the effect of activated oncogenic miRNA or to restore normal expression level in the case or the miRNAs with tumour suppressor role (Figure 3 and Figure 4) [120,125]. Phytochemicals were demonstrated to be able to modify miRNA expression pattern and their mRNA targets, leading to the alteration of different processes involved in cancer (apoptosis cell proliferation and differentiation, angiogenesis, metastasis, and assimilation of drug resistance in cancer [6,118,120,126,127]. The let-7 family of miRNA is listed as one of the key families involved in cancer, where increased expression of this sequences can exercise a decreasing role on tumour growth [128,129,130,131,132,133]. Increased expression of miR-16 and miR-15a had positive effects in chronic lymphocytic leukemia in the form of increased apoptosis [134,135]. The same effect is attributed to miR-34a in pancreatic cells [136]. miR-203 overexpression inhibited pancreatic cell proliferation [137] and tumour growth in a mouse model [138]. miR-17-92 is an oncogenic cluster [139,140,141], but also a tumour suppressor [142]. miR-210 could be an important biomarker, overexpression of this miRNA being associated with poor prognosis in acute myeloid leukaemia (AML) [143] and glioblastoma (GBM) patients [144].

In lung cancer cells from mouse and human origins, EGCG administration increased the expression values for miR-210 through stabilization of HIF-1α (hypoxia-inducible factor 1-α), action that concluded with low proliferation activity for cancer cells [145]. The same treatment in osteosarcoma U2OS cells established that increased expression of miR-126 induce apoptosis acts as a tumour suppressor [146]. miRNA-30b was downregulated by the treatment of HepG2 liver cancer cells with EGCG [147]. EGCG enhanced the curative effect of cisplatin in a BALB/c nude mice model with nonsmall cell lung cancer through downregulation of miR-98-5p, suggesting that hsa-miR-98-5p might be a potential target in clinical cisplatin treatment [148]. EGCG exercised anti-tumour activity upregulating miR-34a/E2F3/Sirt1 in human colon cancer cell lines [149]. The miR-16 lead to apoptosis in HepG2 cells treated with EGCG reducing protein Bcl-2 [150] and in a murine breast cancer model [151]. Chakrabarti et al., in a study of human malignant neuroblastoma cells, showed that EGCG decreased expression of oncogenic miRNAs (miR-92, miR-93 and miR-106b) and increased expression of tumour-suppressor miRNAs (miR-7-1, miR-34a and miR-99a) [152]. EGCG administration in HCC cells (human hepatocellular carcinoma) revealed a pattern of 13 upregulated miRNAs and 48 decreased miRNA [150]. Similar, EGCG treatment determined the downregulation of 5 miRNAs (miR-30b, miR-453, miR-520-e, miR-629, and miR-608) in HepG2 hepatocellular carcinoma cells [153].

Regarding morin and kaempferol, the studies on influence on miRNA are very few in scientific literatures and remain a very interesting field that needs more exploration. Quercetin and kaempferol 3-rutinosides protected CCD-18Co normal colon cells against reactive oxidative species (ROS) by up-regulation of miR-146a, a negative regulator of NFκB activation [154]. Both in vitro and in vivo research activities confirmed that CAPE increases the expression of miR-148a and downregulates the cancer stem cells-like (CSCs-like) characteristics [155].

Genistein treatment leads to the inhibition of oncogenic miR-27a and result in a decreased proliferation rate in ovarian cancer [156] or pancreatic cancer [157]. In other study on MDA-MB-435 and Hs578t cells was observed the antirproliferative effects are via miR-155 and its target genes, *FOXO3*, *PTEN*, casein kinase, and p27 [158]. Both in A-498 line of kidney cancer cells and in the tumours of immunocompromised mice, the photochemical suppressed the expression of miR-21 and reduced tumour formation [159]. The same agent suppressed the proliferation of prostate cancer cells associated with the downregulation of miR-151 [160]. MiR-1260b was significantly downregulated by genistein in prostate cancer tissues [161]. Qin J. et al., demonstrated that in CRC cells it was observed a significantly reduction of miR-95 levels after genistein treatment [162]. In pancreatic malignancies, genistein determined the induction of apoptosis related with miR-223 inhibition [163]. MiR-27a expression was decreased in aggressive melanoma cells simultaneously with the growth of tumours after genistein treatment [164]. It was observed that miR-23b up-regulation after the exposure with a single dose in breast cancer cells might be crucial in patients’ therapeutic strategies [165].

## 6. LncRNAs (Long Non-Coding RNAs) as Targets of Dietary Phytochemicals

As the name suggests, long non-coding RNAs are non-protein coding transcripts longer than 200 nucleotides [188]. LncRNAs do not undergo the process of translation, remaining at the stage of RNA fragments with different lengths that further divide the group into small-non coding RNA and long non-coding RNA [189]. Recent studies demonstrated the potential role of ncRNAs as key regulators of oncogenic and tumour suppressive pathways in cancer, thus leading to different therapeutic strategies targeted on this sequences [190,191].

Administration of phytochemicals acts as suppressor of cell proliferation and invasion, metastasis and stimulators of apoptosis via regulation of lnRNAs expression (Table 3). A novel mechanism of decreasing drug resistance in lung cancer by EGCG, was proved to be via NEAT1 upregulation, leading to an increased response to cisplatin and preventing activation of drug resistance mechanisms in lung cancer [192].

Genistein showed promising results, inhibiting the expression of HOTAIR (HOX transcript antisense RNA) in prostate cancer cell lines, an oncogenic lnRNA that is highly expressed in this type of malignancy. HOTAIR promotes invasiveness and metastasis by silencing the *HOXD* genes and others, playing an important role in cancer advancement [179]. Also, the same phytochemical compound was showed to be a down regulator of HOTAIR in breast cancer, an upregulated predictor of low survival rate, with the same anti-tumour activities as in the case of prostate cancer [193]. Thereby, administration of genistein supports anti-cancer effects, reducing cell proliferation, migration and apoptosis trough down-regulation of oncogenic HOTAIR, in both prostate and breast cancer pathologies [194].

## 7. Multidrug Resistance and Polyphenols Relationship

Multidrug resistance (MDR) in cancer is a major issue regarding the efficiency of anti-cancer drugs, where cells develop an insensitive phenotype and are able to overcome the cytotoxic effects of chemotherapeutics [198]. MDR affects patients with a wide spectrum of malignant pathologies, from blood cancer to solid tumours like lung, ovarian, breast and colorectal cancer. Being one of the reasons for the high mortality within the oncology area, MDR is now in the spotlight for inhibition or reversing strategies. These strategies include also the administration of natural compounds that are able to sensitize cancer cells and contribute to a better response in cancer therapy [199].

MDR is primarily associated with the overexpression of two key membrane “pumps” able to expulse outside of the malignant cell the administrated drug and avoid the associated inhibitory action. The most discussed two molecules in the context of MDR are P-glycoprotein (permeability glycoprotein, Pgd) and so-called multidrug resistance–associated protein (MRP), both representing a target for a wide range of phytochemicals as the latest studies suggests. The research area that comprises the inhibition of the reminded pump have offered to the clinic three generations of inhibitors, but at the current time there are no molecules that could be used as potent and safe inhibition structures due to their negative side effects or incomplete target action. In this sense, natural products are now considered the fourth generation inhibitors with major expectations regarding their ability to reverse MDR. A major positive aspect of these types of molecules is represented by the tolerance of the organism with minimum side effects and also by the diversity of the material that can be used for anticancer strategies [199] and a source for drug discovery of novel natural compounds derivatives with a superior therapeutic effect [200].

Curcumin is an intensively studied polyphenol in cancer scenarios, including the reversal of MDR. It has been shown that this compound is able to restore drug sensitivity in cancer cells that exhibit high amounts of Pgp, MRP1 through direct inhibition of these pumps [201,202,203]. The downside aspect of curcumin is represented by the poor bioavailability of the molecule, but this aspect can be also exceeded by encapsulation of natural compounds inside nanocapsules that increase the administration specificity significantly and also the persistence of the compound inside the organism.

Flavonoids represent one of the biggest classes within the group of plant secondary metabolites with numerous members being studied in the context of MDR [204]. These compounds inhibit the expulsion of cancer drugs outside of the malignant cells mainly by competitive binding to the site responsible for the recognition and recruitment of the specific ligands. This action thus prevents the binding of cytotoxic molecules and implicit their evacuation within the cancer cell. Moreover, polyphenols can act also on the ATP binding sites, molecule that is mandatory for the activity of membrane pumps, or even impair the hydrolysis activity at the domains for nucleotide binding [204,205]. Another inhibitory action of flavonoids on MDR is represented by the modulation of the surface expression of Pgp or MRP, considerably decreasing the number of molecules expressed by the cancer cells and affecting the amount of anti-cancer drugs expulsed from the cells [206].

Considering the latest advances in the context of natural compounds as MDR inhibitors there are increased chances for these structures to become potent inhibitors administrated in the clinic under the form of malignant inhibitory drugs. Moreover, a better approach could be represented by the co-administration of the classical chemotherapeutics along with phytochemicals that could increase their cytotoxicity.

## 8. Conclusions

Phytochemicals are now studied as important regulators of key pathological processes, especially cancer, increasing the awareness regarding the significant contribution of phytochemicals. Furthermore, their action also consists of increased chemotherapeutic sensitivity to numerous treatment agents, improving the overall survival rate in cancer patients. Passing the classical concept as antioxidants associated with natural compounds activity, phytochemicals have been shown to regulate coding, but also non-coding genes with important significance in cancer therapy. In this way, they act as inhibitory compounds towards oncogenic coding and non-coding transcripts and also stimulatory agents that target tumor suppressor transcripts, including miRNAs. Another important aspect consists of the capacity of natural compounds to partially reverse the MDR phenotype of cancer cells, this issue being as one of the major drawbacks in terms of cancer survival rates.

Following these studies, natural agents are now exploited for the development of alternative therapeutic strategies, increasing the action of the conventional treatment schemes in cancer prevention and treatment, through their multitargeting capacity. However, the main obstacle still consists of the reduced bioavailability and stability. In order to overcome this issue, numerous attempts are made in the form of nanostructures, chemical adjustment, and synthetic production and so on. The success of these research activities will significantly contribute to a better manipulation of cancer pathologies, improving the effects of conventional therapies, but also enriching the success of prevention methods.

## Figures and Tables

**Table 1:** Preclinical studies related to the implication of some relevant phytochemicals as antitumoral agents.

Natural Phytochemical	Dose	Preclinical Test	Target Mechanism	Target Gene	Reference
EGCG	0–50 µM	Colon cancer (HT-29 and HCT-116), human embryonic kidney (HEK)-293T cell, Triple negative breast cancer cells (MDA-231)	Apoptosis activation and reduction of cell proliferation via targeting MAPK	*Akt*, *ERK1/2*, *p38*	[56]
	0–200 µM	oral cancer (SSC5)	Reduce cell proliferation, activate apoptosis and autophagy	*BAD*, *BAK*, *FAS*, *IGF1R*, *WNT11*, *ZEB1 CASP8*, *MYC*, and *TP53*	[48]
	0–35 µM	Colorectal cancer cells (LoVo cells, SW480 cells, HT29 cells, and HCT-8 cells) and animal models	induced the apoptosis and affected the cell cycle via Notch signalling	*HES1* and *Notch2*	[56]
	0–160 µM	Inflammatory breast cancer cells (UM-149 and SUM-190)	Inhibit tumoural stemm like comportment	*VEGFD*	[113]
	0–25 µM	Triple negative breast cancer cells	Invasion and angiogenesis	*VEGFA*	[45]
	0–50 µM	Breast cancer and nude mice	Cell proliferation and invasion	*RS/MAPK/p-S6K1*	[55]
	0–100 µM	Gastric cancer cells and nude mice	Cell proliferation, cell cycle, invasion and metastasis	*Wnt/β-catenin*	[114]
Morin	0–350 µM	human leukemic cells (U937 cells)	caspase-dependent apoptosis via intrinsic pathway	*BAX*, *BCL-2*, *cytochrome c*	[64]
	0–400 µM	human colon cancer cells (HCT-116)	ROS generation, extrinsic and intrinsic apoptosis	*Bcl-2* and *IAP* family members, *Fas* and *Akt*	[64]
	0–200 µM	Triple negative breast cancer cells, nude mice	Cell adhesion, EMT, invasion and inhibit lung metastasis	*TNF-α*, *VCAM1* and *N-cadherin*	[67]
	50 µM	Triple negative breast cancer cells, nude mice	EMT, invasion and metastasis	*AKT* and related targets, *MMP-9*	[66]
CAPE	0–100 µM	Breast cancer (MCF-7)	Activate apoptosis and reduce cell proliferation	*NFkB*, *Fas*, *p53*, *Bax* and *JNK*	[79]
	0–50 µM	Oral cancer cells (TW2.6)	Suppress the proliferation, invasion and metastatic potential	*Akt* and *NFkB*	[115]
	0–12 µM	Prostate cancer cells (PC-3)	suppresses the proliferation	*70S6K* and *Akt*	[116]
	0–50 µM	Prostate cancer cells (CRPC)	Cycle arrest and growth inhibition in CRPC cells	*Skp2*, *p53*, *p21Cip1* and *p27Kip1*	[117]
Genistein	0–100 µM	breast cancer cells (MCF-7)	cell proliferation and apoptosis via IGF1R-Akt-Bcl-2 and Bax-mediated pathways	*IGF-1R*, *p-Akt*, *Bcl-2*, and *Bax*	[108]
	10 µmol/L	breast cancer cells (MCF-7)	Cell cycle regulation	*GLIPR1*, *CDC20*, *BUB1*, *MCM2* and *CCNB1*	[109]
	0–50 µM	Colorectal cancer models and orthotopic mouse models	cell invasion and migration, inhibit distant metastasis	*MMP-2* and *FLT4*	[110]
	0–100 µM	colon cancer cells (HCT-116)	Activate mitochondrial apoptosis	*Akt* and *Bax*	[107]
	0.5–10 μmol/L	Prostate cancer cells LAPC-4 and PC-3	Cell proliferation and hormonal receptor	*ER-β*	[106]
Kaempferol	25 μM	Breast cancer cells (MCF-7)	Modulated EMT, inhibit migration, and invasion	*ER*	[95]
	0–100 µM	Bladder cancer	Inhibit cell proliferation	*c-Met/p38*	[94]
	0–50 µM	Lung cancer cells (A549)	Modulated EMT, inhibit migration, and invasion	*TGFβ1*, *SMAD3*, *Akt1*	[96]
	0–100 µM	Oral cancer cells (SCC4)	anti-metastatic effect	*MMP-2* and *TIMP-2*, *c-JUN*, and *ERK1/2*	[111]

**Table 2:** Target miRNAs for different phytochemicals in cancer and their capacity to modulate the expression level of some relevant target genes (overexpression: ↑ or downregulation: ↓).

Phytochemicals	miRNA Transcripts	Expression in Cancer	miRNA Target Gene	Role	References
Epigallocatechin-3-Gallate (EGCG)	miR-16	Hepatocellular carcinoma/↓	*Bcl-2*	Apoptosis induction	[150,166]
	miRNA-330	Breast cancer/↑	*AR*	antagonizes androgen receptor function	[57]
	miR-21	Breast cancer/↓	*AR*	antagonizes androgen receptor function	[57]
	miR-98-5p	Lung cancer/↑	-	Enhance the effect of ciplatin and determines the upregulation of p53 gene	[148]
	miR-30b, miR-453, miR-520-e, miR-629, miR-608	Hepatocellular carcinoma/↑	-	Regulation of inflammation, insulin secretion, glycolysis/gluconeogenesis pathways	[147]
	miR-210	Lung cancer/↓	*HRE*	Disable cell proliferation and suppress cell growth	[167]
	miR-let7b	Melanoma/↓	*67LR*	Inhibits melanoma cells growth via inhibition of HMGA2	[168]
	miR-126	Osteosarcoma/↓	-	Induction of apoptosis and inhibition of cell proliferation	[169]
Morin	No direct studies focused on miRNA expression levels in cancer	Oral tumours, breast, colon and other cancer types/-	-	Anticancer activity via suppression of cell growth and invasion; determines increased sensitivity to chemotherapeutic agents	[66,170,171,172,173]
Caffeic acid phenethyl ester (CAPE)	No direct studies focused on miRNA expression levels in cancer	Lung, prostate and liver cancer/-	-	Anticancer activity through modulation of inflammatory and oxidative stress parameters. Neuroprotective, cardioprotective and hepatoprotective functions	[174,175,176,177]
Genistein	miR-27a	Ovarian cancer/↑	*Sprouty2*	Oncogenic miRNA, promoting tumour growth and migration	[156,178]
	miR-27a	Pancreatic cancer/↑	-	inhibition of miR-27a suppressed cell growth and induced apoptosis as well as inhibited invasion	[157]
	miR-574-3p	Prostate cancer/↓	*RAC1*, *EGFR*, *EP300*	Tumour suppressor miRNA, inhibiting cell proliferation, migration and invasion	[160]
	miR-155	Breast cancer/↑	*OXO3*, *PTEN*, casein kinase, and *p27*	Oncogenic miRNA, promoting tumour growth and migration	[158]
	miR-34a	Prostate cancer/↓	*HOX*	Tumour suppressor miRNA; apoptosis, low invasiveness, decreased cell proliferation	[179]
	miR-1296	Prostate cancer/↓	*MCM*	Inhibits MCM gene family (oncogenes) which was associated with prostate cancer progression	[180]
	miR-221, miR-222	Prostate cancer/↓	*ARH1*	Regulates the expression of ARH1 gene, determining decreased proliferation and invasiveness	[181]
	miR-151	Prostate cancer/↑	*N4BP1*, *ASZ1*, *IL1RAPL1*, *SRY*, *ARHGDIA*	Inhibition of miR-151 was associated with decreased cell migration and invasion, but not proliferation	[182]
	miR-23b-3p	Renal cancer/↑	*PTEN*	Induction of apoptosis in the moment of downregulation	[183]
	miR-1260b	Renal cancer/↑	*sFRP1*, *Dkk2*, *Smad4*	Increased apoptosis and decreased cell proliferation and migration	[184]
Kaempferol	miR-200	Lung cancer/↓	*ZEB1*, *ZEB2*	Inhibitory activity regarding the epithelial-to-mesenchymal transition and migration	[96]
	No other direct studies focused on miRNA expression levels in cancer	Bladder, pancreatic, breast, gastric and prostate cancer/-	-	Inhibitory effects on numerous cancer types, affecting a wide range of genes/pathways: matrix metalloproteinase-9, PTEN, ABCG2, p53, NF-κB, AhR and Nrf2	[40,185,186,187]

**Table 3:** The role of epigallocatechin gallate (EGCG) and genistein on modulation of lncRNA in malignant pathologies (**↑**upregulate the expression level; **↓**downregulate the expression level).

Phytochemicals	ncRNA Transcript	Expression in Cancer	Expression after Natural Treatment	Target Coding or Non-Coding Gene	Role	References
EGCG	NEAT1	Lung cancer/↓	↑	sponging mir-98	EGCG induced CTR1 and enhanced lung cancer cell sensitivity oxaliplatin via hsa-mir-98-5p and NEAT1	[192]
Genistein	HOTAIR	Prostate cancer/↑	↓	*ABL2 SNAIL*, *LAMB3*, *LAMC2*, *MMP9* and *VEGF*	Oncogenic role; regulates invasion and metastasis	[179,195,196]
	HOTAIR	Breast cancer/↑	↓	*HOXD*, *ABL2*, *SNAIL* and *LAMB3*	Oncogenic role; regulates invasion and metastasis	[193,197]
	HOTAIR	Breast cancer/↑	↓	*p-Akt*	Oncogenic role; Inhibit proliferation and activate apoptosis	[194]

## References

[1] Han X., Shen T., Lou H. (2007). Dietary polyphenols and their biological significance. Int. J. Mol. Sci..

[2] Tsao R. (2010). Chemistry and biochemistry of dietary polyphenols. Nutrients.

[3] De la Rosa L.A., Gonzalez-Aguilar G.A., Alvarez-Parrilla E. (2009). Fruit and Vegetable Phytochemicals: Chemistry, Nutritional Value and Stability.

[4] Manach C., Scalbert A., Morand C., Remesy C., Jimenez L. (2004). Polyphenols: Food sources and bioavailability. Am. J. Clin. Nutr..

[5] Russo M., Spagnuolo C., Tedesco I., Russo G.L. (2010). Phytochemicals in cancer prevention and therapy: Truth or dare?. Toxins.

[6] Petric R., Braicu C., Raduly L., Dragos N., Dumitrascu D., Berindan-Negoe I., Zanoaga O., Monroig P. (2015). Phytochemicals modulate carcinogenic signaling pathways in breast and hormone-related cancers. OncoTargets Ther..

[7] Braicu C., Pilecki V., Balacescu O., Irimie A., Neagoe I.B. (2011). The relationships between biological activities and structure of flavan-3-ols. Int. J. Mol. Sci..

[8] Baker M. (2017). Deceptive curcumin offers cautionary tale for chemists. Nature.

[9] Smith T.J. (2011). Green Tea Polyphenols in drug discovery—A success or failure?. Expert Opin. Drug Discov..

[10] Irimie A.I., Braicu C., Cojocneanu-Petric R., Berindan-Neagoe I., Campian R.S. (2015). Novel technologies for oral squamous carcinoma biomarkers in diagnostics and prognostics. Acta Odontol. Scand..

[11] Lam K.S. (2007). New aspects of natural products in drug discovery. Trends Microbiol..

[12] Gu J., Gui Y., Chen L., Yuan G., Lu H.Z., Xu X. (2013). Use of natural products as chemical library for drug discovery and network pharmacology. PLoS ONE.

[13] Bosch R., Philips N., Suárez-Pérez J., Juarranz A., Devmurari A., Chalensouk-Khaosaat J., González S. (2015). Mechanisms of photoaging and cutaneous photocarcinogenesis, and photoprotective strategies with phytochemicals. Antioxidants.

[14] Kusuda M., Hatano T., Yoshida T. (2006). Water-soluble complexes formed by natural polyphenols and bovine serum albumin: Evidence from gel electrophoresis. Biosci. Biotechnol. Biochem..

[15] Isaacs C.E., Wen G.Y., Xu W., Jia J.H., Rohan L., Corbo C., di Maggio V., Jenkins E.C., Hillier S. (2008). Epigallocatechin gallate inactivates clinical isolates of herpes simplex virus. Antimicrob. Agents Chemother..

[16] Stangl V., Dreger H., Stangl K., Lorenz M. (2007). Molecular targets of tea polyphenols in the cardiovascular system. Cardiovasc. Res..

[17] Du G.-J., Zhang Z., Wen X.-D., Yu C., Calway T., Yuan C.-S., Wang C.-Z. (2012). Epigallocatechin gallate (EGCG) is the most effective cancer chemopreventive polyphenol in green tea. Nutrients.

[18] Cai S., Huang J., Wang L., Dong Y., Gong Y., Li J., Li Q., Liu Z., Luo G. (2011). Inhibiting effects of epigallocatechin gallate (EGCG) on the formation of age pigment in vitro and in vivo. J. Med. Plants Res..

[19] Li Y., Tanaka T., Kouno I. (2007). Oxidative coupling of the pyrogallol B-ring with a galloyl group during enzymatic oxidation of epigallocatechin 3-*O*-gallate. Phytochemistry.

[20] Muzolf-Panek M., Gliszczynska-Swiglo A., de Haan L., Aarts J.M., Szymusiak H., Vervoort J.M., Tyrakowska B., Rietjens I.M. (2008). Role of catechin quinones in the induction of EpRE-mediated gene expression. Chem. Res. Toxicol..

[21] Zhang P., Tang Y., Li N.-G., Zhu Y., Duan J.-A. (2014). Bioactivity and chemical synthesis of caffeic acid phenethyl ester and its derivatives. Molecules.

[22] Wang S.H., Chen C.S., Huang S.H., Yu S.H., Lai Z.Y., Huang S.T., Lin C.M. (2009). Hydrophilic ester-bearing chlorogenic acid binds to a novel domain to inhibit xanthine oxidase. Planta Med..

[23] Polkowski K1 M.A. (2000). Biological properties of genistein. A review of in vitro and in vivo data. Acta Pol. Pharm..

[24] Islam N. (2015). Investigation of comparative shielding of morin against oxidative damage by radicals: A DFT study. Cogent Chem..

[25] Brown J. (2003). Enhanced sensitivity of human oral tumours to the flavonol, morin, during cancer progression: Involvement of the Akt and stress kinase pathways. Carcinogenesis.

[26] Lugasi A. (1997). Natural Antioxidants Chemistry, Health Effects, and Applications. Edited byF. Shahidi. VIII and 432 pages, numerous figures and tables. AOCS Press, Champaign, Illinois, 1997. Price: 105.00 U$. Mol. Nutr. Food Res..

[27] Rice-Evans C. (2001). Flavonoid antioxidants. Curr. Med. Chem..

[28] Boyer J., Liu R.H. (2004). Apple phytochemicals and their health benefits. Nutr. J..

[29] Ames B.N., Shigenaga M.K., Hagen T.M. (1993). Oxidants, antioxidants, and the degenerative diseases of aging. Proc. Natl. Acad. Sci. USA.

[30] Hollman P.C.H., Hollman M.B. (1997). Katan, Absorption, metabolism and health effects of dietary flavonoids in man. Biomed. Pharmacother..

[31] Liu R.H. (2003). Health benefits of fruit and vegetables are from additive and synergistic combinations of phytochemicals. Am. J. Clin. Nutr..

[32] Gan R.Y., Li H.B., Sui Z.Q., Corke H. (2016). Absorption, metabolism, anti-cancer effect and molecular targets of epigallocatechin gallate (EGCG): An updated review. Crit. Rev. Food Sci. Nutr..

[33] Seeram N.P., Nair M.G. (2002). Inhibition of lipid peroxidation and structure-activity-related studies of the dietary constituents anthocyanins, anthocyanidins, and catechins. J. Agric. Food Chem..

[34] Wei Y., Chen P., Ling T., Wang Y., Dong R., Zhang C., Zhang L., Han M., Wang D., Wan X. (2016). Certain (−)-epigallocatechin-3-gallate (EGCG) auto-oxidation products (EAOPs) retain the cytotoxic activities of EGCG. Food Chem..

[35] Song S., Huang Y.W., Tian Y., Wang X.J., Sheng J. (2016). Mechanism of action of (−)-epigallocatechin-3-gallate: Auto-oxidation-dependent activation of extracellular signal-regulated kinase 1/2 in Jurkat cells. Chin. J. Natl. Med..

[36] Lee-Hilz Y.Y., Boerboom A.M., Westphal A.H., Berkel W.J., Aarts J.M., Rietjens I.M. (2006). Pro-oxidant activity of flavonoids induces EpRE-mediated gene expression. Chem. Res. Toxicol..

[37] Manson M.M. (2003). Cancer prevention—The potential for diet to modulate molecular signalling. Trends Mol. Med..

[38] Surh Y.-J. (2003). Cancer chemoprevention with dietary phytochemicals. Nat. Rev. Cancer.

[39] Wang L., Chen C. (2013). Emerging applications of metabolomics in studying chemopreventive phytochemicals. AAPS J..

[40] Wang H., Khor T.O., Shu L., Su Z.Y., Fuentes F., Lee J.H., Kong A.N. (2012). Plants vs. cancer: A review on natural phytochemicals in preventing and treating cancers and their druggability. Anticancer Agents Med. Chem..

[41] Pandey K.B., Rizvi S.I. (2009). Plant polyphenols as dietary antioxidants in human health and disease. Oxid. Med. Cell. Longev..

[42] Zhang J., Lei Z., Huang Z., Zhang X., Zhou Y., Luo Z., Zeng W., Su J., Peng C., Chen X. (2016). Epigallocatechin-3-gallate(EGCG) suppresses melanoma cell growth and metastasis by targeting TRAF6 activity. Oncotarget.

[43] Manjegowda M.C., Deb G., Kumar N., Limaye A.M. (2015). Expression profiling of genes modulated by estrogen, EGCG or both in MCF-7 breast cancer cells. Genom. Data.

[44] Braicu C., Gherman C.D., Irimie A., Berindan-Neagoe I. (2013). Epigallocatechin-3-gallate (egcg) inhibits cell proliferation and migratory behaviour of triple negative breast cancer cells. J. Nanosci. Nanotechnol..

[45] Tudoran O., Soritau O., Balacescu O., Balacescu L., Braicu C., Rus M., Gherman C., Virag P., Irimie F., Berindan-Neagoe I. (2012). Early transcriptional pattern of angiogenesis induced by EGCG treatment in cervical tumour cells. J. Cell. Mol. Med..

[46] Irimie A.I., Braicu C., Pileczki V., Petrushev B., Soritau O., Campian R.S., Berindan-Neagoe I. (2016). Knocking down of p53 triggers apoptosis and autophagy, concomitantly with inhibition of migration on SSC-4 oral squamous carcinoma cells. Mol. Cell. Biochem..

[47] Braicu C., Gherman C. (2012). Epigallocatechin gallate induce cell death and apoptosis in triple negative breast cancer cells Hs578T. J. Drug Target..

[48] Irimie A.I., Braicu C., Zanoaga O., Pileczki V., Gherman C., Berindan-Neagoe I., Campian R.S. (2015). Epigallocatechin-3-gallate suppresses cell proliferation and promotes apoptosis and autophagy in oral cancer SSC-4 cells. OncoTargets Ther..

[49] Iriti M., Varoni E. (2013). Chemopreventive potential of flavonoids in oral squamous cell carcinoma in human studies. Nutrients.

[50] Lee U.-L., Choi S.-W. (2011). The chemopreventive properties and therapeutic modulation of green tea polyphenols in oral squamous cell carcinoma. ISRN Oncol..

[51] Zou C., Liu H., Feugang J.M., Hao Z., Chow H.H.S., Garcia F. (2010). Green tea compound in chemoprevention of cervical cancer. Int. J. Gynecol. Cancer.

[52] Shim J.H., Su Z.Y., Chae J.I., Kim D.J., Zhu F., Ma W.Y., Bode A.M., Yang C.S., Dong Z. (2010). Epigallocatechin gallate suppresses lung cancer cell growth through Ras-GTPase-activating protein SH3 domain-binding protein 1. Cancer Prev. Res..

[53] Jin H., Gong W., Zhang C., Wang S. (2013). Epigallocatechin gallate inhibits the proliferation of colorectal cancer cells by regulating Notch signaling. OncoTargets Ther..

[54] Albrecht D.S., Clubbs E.A., Ferruzzi M., Bomser J.A. (2008). Epigallocatechin-3-gallate (EGCG) inhibits PC-3 prostate cancer cell proliferation via MEK-independent ERK1/2 activation. Chemi. Biol. Interact..

[55] Zhou Y., Tang J., Du Y., Ding J., Liu J.Y. (2016). The green tea polyphenol EGCG potentiates the antiproliferative activity of sunitinib in human cancer cells. Tumour Biol..

[56] Cerezo-Guisado M.I., Zur R., Lorenzo M.J., Risco A., Martin-Serrano M.A., Alvarez-Barrientos A., Cuenda A., Centeno F. (2015). Implication of Akt, ERK1/2 and alternative p38MAPK signalling pathways in human colon cancer cell apoptosis induced by green tea EGCG. Food Chem. Toxicol..

[57] Siddiqui I.A., Asim M., Hafeez B.B., Adhami V.M., Tarapore R.S., Mukhtar H. (2011). Green tea polyphenol EGCG blunts androgen receptor function in prostate cancer. FASEB J..

[58] Lee Y.H., Kwak J., Choi H.K., Choi K.C., Kim S., Lee J., Jun W., Park H.J., Yoon H.G. (2012). EGCG suppresses prostate cancer cell growth modulating acetylation of androgen receptor by anti-histone acetyltransferase activity. Int. J. Mol. Med..

[59] Kawabata K., Tanaka T., Honjo S., Kakumoto M., Hara A., Makita H., Tatematsu N., Ushida J., Tsuda H., Mori H. (1999). Chemopreventive effect of dietary flavonoid morin on chemically induced rat tongue carcinogenesis. Int. J. Cancer.

[60] Karimi R., Parivar K., Roudbari N.H., Sadeghi S.V., Hashemi M., Hayat P. (2013). Anti-proliferative and apoptotic effects of morin in human Leukemia cell lines (HUT-78). Int. J. Cell. Mol. Biotechnol..

[61] Hsiang C.Y., Wu S.L., Ho T.Y. (2005). Morin inhibits 12-*O*-tetradecanoylphorbol-13-acetate-induced hepatocellular transformation via activator protein 1 signaling pathway and cell cycle progression. Biochem. Pharmacol..

[62] Kuo H.M., Chang L.S., Lin Y.L., Lu H.F., Yang J.S., Lee J.H., Chung J.G. (2007). Morin inhibits the growth of human leukemia HL-60 cells via cell cycle arrest and induction of apoptosis through mitochondria dependent pathway. Anticancer Res..

[63] Kondath S., Raghavan B.S., Anantanarayanan R., Rajaram R. (2014). Synthesis and characterisation of morin reduced gold nanoparticles and its cytotoxicity in MCF-7 cells. Chem. Biol. Int..

[64] Park C., Lee W.S., Go S., Nagappan A., Han M.H., Hong S.H., Kim G.S., Kim G.Y., Kwon T.K., Ryu C.H. (2015). Morin, a flavonoid from moraceae, induces apoptosis by induction of BAD protein in human Leukemic cells. Int. J. Mol. Sci..

[65] Hyun H.B., Lee W.S., Go S.I., Nagappan A., Park C., Han M.H., Hong S.H., Kim G., Kim G.Y., Cheong J. (2015). The flavonoid morin from Moraceae induces apoptosis by modulation of Bcl-2 family members and Fas receptor in HCT 116 cells. Int. J. Oncol..

[66] Jin H., Lee W.S., Eun S.Y., Jung J.H., Park H.S., Kim G., Choi Y.H., Ryu C.H., Jung J.M., Hong S.C. (2014). Morin, a flavonoid from Moraceae, suppresses growth and invasion of the highly metastatic breast cancer cell line MDA-MB231 partly through suppression of the Akt pathway. Int. J. Oncol..

[67] Lee J., Jin H., Lee W.S., Nagappan A., Choi Y.H., Kim G.S., Jung J., Ryu C.H., Shin S.C., Hong S.C. (2016). Morin, a flavonoid from moraceae, inhibits cancer cell adhesion to endothelial cells and EMT by downregulating VCAM1 and ncadherin. Asian Pac. J. Cancer Prev..

[68] Son S., Lewis B.A. (2002). Free radical scavenging and antioxidative activity of caffeic acid amide and ester analogues: Structure−activity relationship. J. Agric. Food Chem..

[69] Wu J., Omene C., Karkoszka J., Bosland M., Eckard J., Klein C.B., Frenkel K. (2011). Caffeic acid phenethyl ester (CAPE), derived from a honeybee product propolis, exhibits a diversity of anti-tumor effects in pre-clinical models of human breast cancer. Cancer Lett..

[70] Rzepecka-Stojko A., Kabała-Dzik A., Moździerz A., Kubina R., Wojtyczka R., Stojko R., Dziedzic A., Jastrzębska-Stojko Ż., Jurzak M., Buszman E. (2015). Caffeic acid phenethyl ester and ethanol extract of propolis induce the complementary cytotoxic effect on triple-negative breast cancer cell lines. Molecules.

[71] Cho M.S., Park W.S., Jung W.-K., Qian Z.-J., Lee D.-S., Choi J.-S., Lee D.-Y., Park S.-G., Seo S.-K., Kim H.-J. (2014). Caffeic acid phenethyl ester promotes anti-inflammatory effects by inhibiting MAPK and NF-κB signaling in activated HMC-1 human mast cells. Pharm. Biol..

[72] Altuntaş A., Yılmaz H.R., Altuntaş A., Uz E., Demir M., Gökçimen A., Aksu O., Bayram D.Ş., Sezer M.T. (2014). Caffeic acid phenethyl ester protects against amphotericin B induced nephrotoxicity in rat model. BioMed Res. Int..

[73] Zhou K., Li X., Du Q., Li D., Hu M., Yang X., Jiang Q., Li Z. (2014). A CAPE analogue as novel antiplatelet agent efficiently inhibits collagen-induced platelet aggregation. Pharmazie.

[74] Gherman C., Braicu O.L., Zanoaga O., Jurj A., Pileczki V., Maralani M., Drigla F., Braicu C., Budisan L., Achimas-Cadariu P. (2016). Caffeic acid phenethyl ester activates pro-apoptotic and epithelial-mesenchymal transition-related genes in ovarian cancer cells A2780 and A2780*cis*. Mol. Cell. Biochem..

[75] Xiang D., Wang D., He Y., Xie J., Zhong Z., Li Z., Xie J. (2006). Caffeic acid phenethyl ester induces growth arrest and apoptosis of colon cancer cells via the β-catenin/T-cell factor signaling. Anticancer Drugs.

[76] Omene C.O., Wu J., Frenkel K. (2012). Caffeic acid phenethyl ester (CAPE) derived from propolis, a honeybee product, inhibits growth of breast cancer stem cells. Investig. New Drugs.

[77] Lin H.-P., Jiang S.S., Chuu C.-P. (2012). Caffeic acid phenethyl ester causes p21Cip1 induction, Akt signaling reduction, and growth inhibition in PC-3 human prostate cancer cells. PLoS ONE.

[78] Kuo Y.-Y., Jim W.-T., Su L.-C., Chung C.-J., Lin C.-Y., Huo C., Tseng J.-C., Huang S.-H., Lai C.-J., Chen B.-C. (2015). Caffeic acid phenethyl ester is a potential therapeutic agent for oral cancer. Int. J. Mol. Sci..

[79] Watabe M., Hishikawa K., Takayanagi A., Shimizu N., Nakaki T. (2004). Caffeic acid phenethyl ester induces apoptosis by inhibition of NFκB and activation of Fas in human breast cancer MCF-7 cells. J. Biol. Chem..

[80] Hsu T.-H., Chu C.-C., Hung M.-W., Lee H.-J., Hsu H.-J., Chang T.-C. (2013). Caffeic acid phenethyl ester induces E2F-1-mediated growth inhibition and cell-cycle arrest in human cervical cancer cells. FEBS J..

[81] Huang W.-W., Chiu Y.-J., Fan M.-J., Lu H.-F., Yeh H.-F., Li K.-H., Chen P.-Y., Chung J.-G., Yang J.-S. (2010). Kaempferol induced apoptosis via endoplasmic reticulum stress and mitochondria-dependent pathway in human osteosarcoma U-2 OS cells. Mol. Nutr. Food Res..

[82] Kim B.-W., Lee E.-R., Min H.-M., Jeong H.-S., Ahn J.-Y., Kim J.-H., Choi H.-Y., Choi H., Kim E.Y., Park S.P. (2008). Sustained ERK activation is involved in the kaempferol-induced apoptosis of breast cancer cells and is more evident under 3-D culture condition. Cancer Biol. Ther..

[83] Lee K.M., Lee D.E., Seo S.K., Hwang M.K., Heo Y.S., Lee K.W., Lee H.J. (2010). Phosphatidylinositol 3-kinase, a novel target molecule for the inhibitory effects of kaempferol on neoplastic cell transformation. Carcinogenesis.

[84] Ackland M.L., van de Waarsenburg S., Jones R. (2005). Synergistic antiproliferative action of the flavonols quercetin and kaempferol in cultured human cancer cell lines. In Vivo.

[85] Luo H., Daddysman M.K., Rankin G.O., Jiang B.-H., Chen Y.C. (2010). Kaempferol enhances cisplatin’s effect on ovarian cancer cells through promoting apoptosis caused by down regulation of cMyc. Cancer Cell. Int..

[86] Zhang Y., Chen A.Y., Li M., Chen C., Yao Q. (2008). *Ginkgo biloba* extract kaempferol inhibits cell proliferation and induces apoptosis in pancreatic cancer cells. J. Surg. Res..

[87] Lin C.-W., Chen P.-N., Chen M.-K., Yang W.-E., Tang C.-H., Yang S.-F., Hsieh Y.-S. (2013). Kaempferol reduces matrix metalloproteinase-2 expression by down-regulating ERK1/2 and the activator protein-1 signaling pathways in oral cancer cells. PLoS ONE.

[88] Huang W.W., Tsai S.C., Peng S.F., Lin M.W., Chiang J.H., Chiu Y.J., Fushiya S., Tseng M.T., Yang J.S. (2013). Kaempferol induces autophagy through AMPK and AKT signaling molecules and causes G2/M arrest via downregulation of CDK1/cyclin B in SK-HEP-1 human hepatic cancer cells. Int. J. Oncol..

[89] Choi E.J., Ahn W.S. (2008). Kaempferol induced the apoptosis via cell cycle arrest in human breast cancer MDA-MB-453 cells. Nutr. Res. Pract..

[90] Tsiklauri L., An G., Ruszaj D.M., Alaniya M., Kemertelidze E., Morris M.E. (2011). Simultaneous determination of the flavonoids robinin and kaempferol in human breast cancer cells by liquid chromatography-tandem mass spectrometry. J. Pharm. Biom. Anal..

[91] Jeong J.C., Kim M.S., Kim T.H., Kim Y.K. (2008). Kaempferol induces cell death through ERK and Akt-dependent down-regulation of XIAP and survivin in human glioma cells. Neurochem. Res..

[92] Marfe G., Tafani M., Indelicato M., Sinibaldi-Salimei P., Reali V., Pucci B., Fini M., Russo M.A. (2009). Kaempferol induces apoptosis in two different cell lines via Akt inactivation, Bax and SIRT3 activation, and mitochondrial dysfunction. J. Cell. Biochem..

[93] Xie F., Su M., Qiu W., Zhang M., Guo Z., Su B., Liu J., Li X., Zhou L. (2013). Kaempferol promotes apoptosis in human bladder cancer cells by inducing the tumor suppressor, PTEN. Int. J. Mol. Sci..

[94] Dang Q., Song W., Xu D., Ma Y., Li F., Zeng J., Zhu G., Wang X., Chang L.S., He D., Li L. (2015). Kaempferol suppresses bladder cancer tumor growth by inhibiting cell proliferation and inducing apoptosis. Mol. Carcinog..

[95] Lee G.A., Choi K.C., Hwang K.A. (2017). Kaempferol, a phytoestrogen, suppressed triclosan-induced epithelial-mesenchymal transition and metastatic-related behaviors of MCF-7 breast cancer cells. Environ. Toxicol. Pharm..

[96] Jo E., Park S.J., Choi Y.S., Jeon W.K., Kim B.C. (2015). Kaempferol suppresses transforming growth factor-β1-induced epithelial-to-mesenchymal transition and migration of A549 lung cancer cells by inhibiting Akt1-mediated phosphorylation of Smad3 at Threonine-179. Neoplasia.

[97] Li Y., Kong D., Bao B., Ahmad A., Sarkar F.H. (2011). Induction of cancer cell death by isoflavone: The role of multiple signaling pathways. Nutrients.

[98] Zhu J., Zhang C., Qing Y., Cheng Y., Jiang X., Li M., Yang Z., Wang D. (2015). Genistein induces apoptosis by stabilizing intracellular p53 protein through an APE1-mediated pathway. Free Radic. Biol. Med..

[99] Mahmoud A.M., Zhu T., Parray A., Siddique H.R., Yang W., Saleem M., Bosland M.C. (2013). Differential effects of genistein on prostate cancer cells depend on mutational status of the androgen receptor. PLoS ONE.

[100] Zhang D., Tai Y.-C., Wong C.-H.S., Tai L.-K., Koay E.S.-C., Chen C.-S. (2007). Molecular response of leukemia HL-60 cells to genistein treatment, a proteomics study. Leuk. Res..

[101] Narasimhan K., Lee Y.M., Lim T.K., Port S.A., Han J.-H., Chen C.-S., Lin Q. (2015). Genistein exerts anti-leukemic effects on genetically different acute myeloid leukemia cell lines by inhibiting protein synthesis and cell proliferation while inducing apoptosis—Molecular insights from an iTRAQ™ quantitative proteomics study. Oncoscience.

[102] Pavese J.M., Krishna S.N., Bergan R.C. (2014). Genistein inhibits human prostate cancer cell detachment, invasion, and metastasis. Am. J. Clin. Nutr..

[103] Pons D.G., Nadal-Serrano M., Torrens-Mas M., Oliver J., Roca P. (2015). The phytoestrogen genistein affects breast cancer cells treatment depending on the ERα/ERβ ratio. J. Cell. Biochem..

[104] Chen W.F., Wong M.S. (2004). Genistein enhances insulin-like growth factor signaling pathway in human breast cancer (MCF-7) cells. J. Clin. Endocrinol. Metab..

[105] Karsli-Ceppioglu S., Ngollo M., Judes G., Penault-Llorca F., Bignon Y.-J., Guy L., Bernard-Gallon D. (2015). The Role of Soy Phytoestrogens on Genetic and Epigenetic Mechanisms of Prostate Cancer. Mechanism of the Anticancer Effect of Phytochemicals.

[106] Russo M., Russo G.L., Daglia M., Kasi P.D., Ravi S., Nabavi S.F., Nabavi S.M. (2016). Understanding genistein in cancer: The “good” and the “bad” effects: A review. Food Chem..

[107] Qin J., Teng J., Zhu Z., Chen J., Huang W.J. (2016). Genistein induces activation of the mitochondrial apoptosis pathway by inhibiting phosphorylation of Akt in colorectal cancer cells. Pharm. Biol..

[108] Chen J., Duan Y., Zhang X., Ye Y., Ge B., Chen J. (2015). Genistein induces apoptosis by the inactivation of the IGF-1R/p-Akt signaling pathway in MCF-7 human breast cancer cells. Food Funct..

[109] Zhang L., Yang B., Zhou K., Li H., Li D., Gao H., Zhang T., Wei D., Li Z., Diao Y. (2015). Potential therapeutic mechanism of genistein in breast cancer involves inhibition of cell cycle regulation. Mol. Med. Rep..

[110] Xiao X., Liu Z., Wang R., Wang J., Zhang S., Cai X., Wu K., Bergan R.C., Xu L., Fan D. (2015). Genistein suppresses FLT4 and inhibits human colorectal cancer metastasis. Oncotarget.

[111] Chen H.J., Lin C.M., Lee C.Y., Shih N.C., Peng S.F., Tsuzuki M., Amagaya S., Huang W.W., Yang J.S. (2013). Kaempferol suppresses cell metastasis via inhibition of the ERK-p38-JNK and AP-1 signaling pathways in U-2 OS human osteosarcoma cells. Oncol. Rep..

[112] Mahmoud A.M., Al-Alem U., Ali M.M., Bosland M.C. (2015). Genistein increases estrogen receptor beta expression in prostate cancer via reducing its promoter methylation. J. Steroid Biochem. Mol. Biol..

[113] Mineva N.D., Paulson K.E., Naber S.P., Yee A.S., Sonenshein G.E. (2013). Epigallocatechin-3-gallate inhibits stem-like inflammatory breast cancer cells. PLoS ONE.

[114] Yang C., Du W., Yang D. (2016). Inhibition of green tea polyphenol EGCG((−)-epigallocatechin-3-gallate) on the proliferation of gastric cancer cells by suppressing canonical Wnt/β-catenin signalling pathway. Int. J. Food Sci. Nutr..

[115] Kuo Y.Y., Lin H.P., Huo C., Su L.C., Yang J., Hsiao P.H., Chiang H.C., Chung C.J., Wang H.D., Chang J.Y. (2013). Caffeic acid phenethyl ester suppresses proliferation and survival of TW2.6 human oral cancer cells via inhibition of Akt signaling. Int. J. Mol. Sci..

[116] Chuu C.P., Lin H.P., Ciaccio M.F., Kokontis J.M., Hause R.J., Hiipakka R.A., Liao S., Jones R.B. (2012). Caffeic acid phenethyl ester suppresses the proliferation of human prostate cancer cells through inhibition of p70S6K and Akt signaling networks. Cancer Prev. Res..

[117] Lin H.P., Lin C.Y., Huo C., Hsiao P.H., Su L.C., Jiang S.S., Chan T.M., Chang C.H., Chen L.T., Kung H.J. (2015). Caffeic acid phenethyl ester induced cell cycle arrest and growth inhibition in androgen-independent prostate cancer cells via regulation of Skp2, p53, p21Cip1 and p27Kip1. Oncotarget.

[118] Braicu C., Tomuleasa C., Monroig P., Cucuianu A., Berindan-Neagoe I., Calin G.A. (2015). Exosomes as divine messengers: Are they the Hermes of modern molecular oncology?. Cell Death Differ..

[119] Berindan-Neagoe I., Calin G.A. (2014). Molecular pathways: MicroRNAs, cancer cells, and microenvironment. Clin. Cancer Res..

[120] Berindan-Neagoe I., Pdel C.M., Pasculli B., Calin G.A. (2014). MicroRNAome genome: A treasure for cancer diagnosis and therapy. CA Cancer J. Clin..

[121] Srivastava S.K., Arora S., Averett C., Singh S., Singh A.P. (2015). Modulation of microRNAs by phytochemicals in cancer: Underlying mechanisms and translational significance. BioMed Res. Int..

[122] Thakur V.S., Deb G., Babcook M.A., Gupta S. (2014). Plant phytochemicals as epigenetic modulators: Role in cancer chemoprevention. AAPS J..

[123] Masika J., Hescheler J., Liang H., Zhao Y. (2016). Modulation of miRNAs by natural agents: Nature’s way of dealing with cancer. RNA Discov..

[124] Biersack B. (2016). Non-coding RNA/microRNA-modulatory dietary factors and natural products for improved cancer therapy and prevention: Alkaloids, organosulfur compounds, aliphatic carboxylic acids and water-soluble vitamins. Non-Coding RNA Res..

[125] Shankar S., Kumar D., Srivastava R.K. (2013). Epigenetic modifications by dietary phytochemicals: Implications for personalized nutrition. Pharmacol. Ther..

[126] Naselli F., Belshaw N.J., Gentile C., Tutone M., Tesoriere L., Livrea M.A., Caradonna F. (2015). Phytochemical indicaxanthin inhibits colon cancer cell growth and affects the DNA methylation status by influencing epigenetically modifying enzyme expression and activity. J. Nutr. Nutr..

[127] Nakazawa T., Hayashi K., Naitoh I., Miyabe K., Shimizu S., Kondo H., Nishi Y., Yoshida M., Umemura S., Hori Y. (2015). Chemopreventive effect of resveratrol and apocynin on pancreatic carcinogenesis via modulation of nuclear phosphorylated GSK3β and ERK1/2. Oncotarget.

[128] Johnson S.M., Grosshans H., Shingara J., Byrom M., Jarvis R., Cheng A., Labourier E., Reinert K.L., Brown D., Slack F.J. (2005). RAS is regulated by the let-7 microRNA family. Cell.

[129] Barh D., Malhotra R., Ravi B., Sindhurani P. (2010). Microrna let-7: An emerging next-generation cancer therapeutic. Curr. Oncol..

[130] Takamizawa J. (2004). Reduced expression of the let-7 microRNAs in human lung cancers in association with shortened postoperative survival. Cancer Res..

[131] Brueckner B., Stresemann C., Kuner R., Mund C., Musch T., Meister M., Sultmann H., Lyko F. (2007). The Human let-7a-3 locus contains an epigenetically regulated microRNA gene with oncogenic function. Cancer Res..

[132] Xia X.-M. (2010). Clinical significance and the correlation of expression between Let-7 and K-ras in non-small cell lung cancer. Oncol. Lett..

[133] Zhao Y., Deng C., Wang J., Xiao J., Gatalica Z., Recker R.R., Xiao G.G. (2011). Let-7 family miRNAs regulate estrogen receptor α signaling in estrogen receptor positive breast cancer. Breast Cancer Res. Treat..

[134] Cimmino A., Calin G.A., Fabbri M., Iorio M.V., Ferracin M., Shimizu M., Wojcik S.E., Aqeilan R.I., Zupo S., Dono M. (2005). miR-15 and miR-16 induce apoptosis by targeting BCL2. Proc. Natl. Acad. Sci. USA.

[135] Aqeilan R.I., Calin G.A., Croce C.M. (2009). miR-15a and miR-16–1 in cancer: Discovery, function and future perspectives. Cell Death Differ..

[136] Chang T.-C., Wentzel E.A., Kent O.A., Ramachandran K., Mullendore M., Lee K.H., Feldmann G., Yamakuchi M., Ferlito M., Lowenstein C.J. (2007). Transactivation of miR-34a by p53 broadly influences gene expression and promotes apoptosis. Mol. Cell.

[137] Xu L. (2013). miR-203 regulates the proliferation, apoptosis and cell cycle progression of pancreatic cancer cells by targeting survivin. Mol. Med. Rep..

[138] Sonkoly E., Lovén J., Xu N., Meisgen F., Wei T., Brodin P., Jaks V., Kasper M., Shimokawa T., Harada M. (2012). MicroRNA-203 functions as a tumor suppressor in basal cell carcinoma. Oncogenesis.

[139] He L., Thomson J.M., Hemann M.T., Hernando-Monge E., Mu D., Goodson S., Powers S., Cordon-Cardo C., Lowe S.W., Hannon G.J. (2005). A microRNA polycistron as a potential human oncogene. Nature.

[140] Rinaldi A., Poretti G., Kwee I., Zucca E., Catapano C.V., Tibiletti M.G., Bertoni F. (2007). Concomitant MYC and microRNA cluster miR-17–92 (C13orf25) amplification in human mantle cell lymphoma. Leuk. Lymphoma.

[141] Mendell J.T. (2008). miRiad roles for the miR-17–92 cluster in development and disease. Cell.

[142] Zhang L., Huang J., Yang N., Greshock J., Megraw M.S., Giannakakis A., Liang S., Naylor T.L., Barchetti A., Ward M.R. (2006). microRNAs exhibit high frequency genomic alterations in human cancer. Proc. Natl. Acad. Sci. USA.

[143] Tang R., Liang L., Luo D., Feng Z., Huang Q., He R., Gan T., Yang L., Chen G. (2015). Downregulation of miR-30a is associated with poor prognosis in lung cancer. Med. Sci. Monit..

[144] Zhang S., Lai N., Liao K., Sun J., Lin Y. (2015). microRNA-210 regulates cell proliferation and apoptosis by targeting regulator of differentiation 1 in glioblastoma cells. Folia Neuropathol..

[145] Thomas R., Kim M.H. (2005). Epigallocatechin gallate inhibits HIF-1α degradation in prostate cancer cells. Biochem. Biophys. Res. Commun..

[146] Zhu K., Wang W. (2016). Green tea polyphenol EGCG suppresses osteosarcoma cell growth through upregulating miR-1. Tumour Biol..

[147] Arola-Arnal A., Blade C. (2011). Proanthocyanidins modulate microRNA expression in human HepG2 cells. PLoS ONE.

[148] Zhou D.H., Wang X., Feng Q. (2014). EGCG enhances the efficacy of cisplatin by downregulating hsa-miR-98-5p in NSCLC A549 cells. Nutr. Cancer.

[149] Kumazaki M., Noguchi S., Yasui Y., Iwasaki J., Shinohara H., Yamada N., Akao Y. (2013). Anti-cancer effects of naturally occurring compounds through modulation of signal transduction and miRNA expression in human colon cancer cells. J. Nutr. Biochem..

[150] Tsang W.P., Kwok T.T. (2010). Epigallocatechin gallate up-regulation of miR-16 and induction of apoptosis in human cancer cells. J. Nutr. Biochem..

[151] Jang J.-Y., Lee J.-K., Jeon Y.-K., Kim C.-W. (2013). Exosome derived from epigallocatechin gallate treated breast cancer cells suppresses tumor growth by inhibiting tumor-associated macrophage infiltration and M2 polarization. BMC Cancer.

[152] Chakrabarti M., Khandkar M., Banik N.L., Ray S.K. (2012). Alterations in expression of specific microRNAs by combination of 4-HPR and EGCG inhibited growth of human malignant neuroblastoma cells. Brain Res..

[153] Farooqi A.A., Gadaleta C.D., Ranieri G., Fayyaz S., Marech I. (2016). New frontiers in promoting TRAIL-mediated cell death: Focus on natural sensitizers, miRNAs, and nanotechnological advancements. Cell Biochem. Biophys..

[154] Noratto G.D., Kim Y., Talcott S.T., Mertens-Talcott S.U. (2011). Flavonol-rich fractions of yaupon holly leaves (*Ilex vomitoria*, *Aquifoliaceae*) induce microRNA-146a and have anti-inflammatory and chemopreventive effects in intestinal myofribroblast CCD-18Co cells. Fitoterapia.

[155] Li Y., Jiang F., Chen L., Yang Y., Cao S., Ye Y., Wang X., Mu J., Li Z., Li L. (2015). Blockage of TGFβ-SMAD2 by demethylation-activated miR-148a is involved in caffeic acid-induced inhibition of cancer stem cell-like properties in vitro and in vivo. FEBS Open Bio.

[156] Xu L., Xiang J., Shen J., Zou X., Zhai S., Yin Y., Li P., Wang X., Sun Q. (2013). Oncogenic microRNA-27a is a target for genistein in ovarian cancer cells. Anticancer Agents Med. Chem..

[157] Xia J., Cheng L., Mei C., Ma J., Shi Y., Zeng F., Wang Z., Wang Z. (2014). Genistein inhibits cell growth and invasion through regulation of miR-27a in pancreatic cancer cells. Curr. Pharm. Des..

[158] De la Parra C., Castillo-Pichardo L., Cruz-Collazo A., Cubano L., Redis R., Calin G.A., Dharmawardhane S. (2016). Soy isoflavone genistein-mediated downregulation of miR-155 contributes to the anticancer effects of genistein. Nutr. Cancer.

[159] Zaman M.S., Shahryari V., Deng G., Thamminana S., Saini S., Majid S., Chang I., Hirata H., Ueno K., Yamamura S. (2012). Up-regulation of microRNA-21 correlates with lower kidney cancer survival. PLoS ONE.

[160] Chiyomaru T., Yamamura S., Fukuhara S., Hidaka H., Majid S., Saini S., Arora S., Deng G., Shahryari V., Chang I. (2013). Genistein up-regulates tumor suppressor microRNA-574-3p in prostate cancer. PLoS ONE.

[161] Hirata H., Hinoda Y., Shahryari V., Deng G., Tanaka Y., Tabatabai Z.L., Dahiya R. (2014). Genistein downregulates onco-miR-1260b and upregulates sFRP1 and Smad4 via demethylation and histone modification in prostate cancer cells. Br. J. Cancer.

[162] Qin J., Chen J.X., Zhu Z., Teng J.A. (2015). Genistein inhibits human colorectal cancer growth and suppresses miR-95, Akt and SGK1. Cell. Physiol. Biochem..

[163] Ma J., Liu H., Cheng L., Zhang J., Shi Y., Zeng F., Miele L., Sarkar F.H., Xia J., Wang Z. (2013). Genistein down-regulates miR-223 expression in pancreatic cancer cells. Curr. Drug. Targets.

[164] Sun Q., Cong R., Yan H., Gu H., Zeng Y., Liu N., Chen J., Wang B. (2009). Genistein inhibits growth of human uveal melanoma cells and affects microRNA-27a and target gene expression. Oncol. Rep..

[165] Avci C.B., Susluer S.Y., Caglar H.O., Balci T., Aygunes D., Dodurga Y., Gunduz C. (2015). Genistein-induced miR-23b expression inhibits the growth of breast cancer cells. Contemp. Oncol..

[166] Wu W.L., Wang W.Y., Yao W.Q., Li G.D. (2015). Suppressive effects of microRNA-16 on the proliferation, invasion and metastasis of hepatocellular carcinoma cells. Int. J. Mol. Med..

[167] Wang H., Bian S., Yang C.S. (2011). Green tea polyphenol EGCG suppresses lung cancer cell growth through upregulating miR-210 expression caused by stabilizing HIF-1α. Carcinogenesis.

[168] Yamada S., Tsukamoto S., Huang Y., Makio A., Kumazoe M., Yamashita S., Tachibana H. (2016). Epigallocatechin-3-*O*-gallate up-regulates microRNA-let-7b expression by activating 67-kDa laminin receptor signaling in melanoma cells. Sci. Rep..

[169] Jiang L., Tao C., He A., He X. (2014). Overexpression of miR-126 sensitizes osteosarcoma cells to apoptosis induced by epigallocatechin-3-gallate. World J. Surg. Oncol..

[170] Naso L.G., Lezama L., Rojo T., Etcheverry S.B., Valcarcel M., Roura M., Salado C., Ferrer E.G., Williams P.A. (2013). Biological evaluation of morin and its new oxovanadium(IV) complex as antioxidant and specific anti-cancer agents. Chem. Biol. Interact..

[171] Kumar V.K., Vennila S., Nalini N. (2009). Modifying effects of morin on the development of aberrant crypt foci and bacterial enzymes in experimental colon cancer. Food Chem. Toxicol..

[172] Jung J.S., Choi M.J., Lee Y.Y., Moon B.I., Park J.S., Kim H.S. (2017). Suppression of lipopolysaccharide-induced neuroinflammation by morin via MAPK, PI3K/Akt, and PKA/HO-1 signaling pathway modulation. J. Agric. Food Chem..

[173] Hussain J., Ali L., Khan A.L., Rehman N.U., Jabeen F., Kim J.S., Al-Harrasi A. (2014). Isolation and bioactivities of the flavonoids morin and morin-3-*O*-β-d-glucopyranoside from *Acridocarpus orientalis*—A wild Arabian medicinal plant. Molecules.

[174] Stagos D., Amoutzias G.D., Matakos A., Spyrou A., Tsatsakis A.M., Kouretas D. (2012). Chemoprevention of liver cancer by plant polyphenols. Food Chem. Toxicol..

[175] Lall R.K., Syed D.N., Adhami V.M., Khan M.I., Mukhtar H. (2015). Dietary polyphenols in prevention and treatment of prostate cancer. Int. J. Mol. Sci..

[176] Tolba M.F., Azab S.S., Khalifa A.E., Abdel-Rahman S.Z., Abdel-Naim A.B. (2013). Caffeic acid phenethyl ester, a promising component of propolis with a plethora of biological activities: A review on its anti-inflammatory, neuroprotective, hepatoprotective, and cardioprotective effects. IUBMB Life.

[177] Milenkovic D., Deval C., Gouranton E., Landrier J.F., Scalbert A., Morand C., Mazur A. (2012). Modulation of miRNA expression by dietary polyphenols in apoE deficient mice: A new mechanism of the action of polyphenols. PLoS ONE.

[178] Taylor C.K., Levy R.M., Elliott J.C., Burnett B.P. (2009). The effect of genistein aglycone on cancer and cancer risk: A review of in vitro, preclinical, and clinical studies. Nutr. Rev..

[179] Chiyomaru T., Yamamura S., Fukuhara S., Yoshino H., Kinoshita T., Majid S., Saini S., Chang I., Tanaka Y., Enokida H. (2013). Genistein inhibits prostate cancer cell growth by targeting miR-34a and oncogenic HOTAIR. PLoS ONE.

[180] Phuah N.H., Nagoor N.H. (2014). Regulation of microRNAs by natural agents: New strategies in cancer therapies. BioMed Res. Int..

[181] Chen Y., Zaman M.S., Deng G., Majid S., Saini S., Liu J., Tanaka Y., Dahiya R. (2011). MicroRNAs 221/222 and genistein-mediated regulation of ARHI tumor suppressor gene in prostate cancer. Cancer Prev. Res..

[182] Chiyomaru T., Yamamura S., Zaman M.S., Majid S., Deng G., Shahryari V., Saini S., Hirata H., Ueno K., Chang I. (2012). Genistein suppresses prostate cancer growth through inhibition of oncogenic microRNA-151. PLoS ONE.

[183] Zaman M.S., Thamminana S., Shahryari V., Chiyomaru T., Deng G., Saini S., Majid S., Fukuhara S., Chang I., Arora S. (2012). Inhibition of *PTEN* gene expression by oncogenic miR-23b-3p in renal cancer. PLoS ONE.

[184] Hirata H., Ueno K., Nakajima K., Tabatabai Z.L., Hinoda Y., Ishii N., Dahiya R. (2013). Genistein downregulates onco-miR-1260b and inhibits Wnt-signalling in renal cancer cells. Br. J. Cancer.

[185] Azevedo C., Correia-Branco A., Araujo J.R., Guimaraes J.T., Keating E., Martel F. (2015). The chemopreventive effect of the dietary compound kaempferol on the MCF-7 human breast cancer cell line is dependent on inhibition of glucose cellular uptake. Nutr. Cancer.

[186] Li C., Zhao Y., Yang D., Yu Y., Guo H., Zhao Z., Zhang B., Yin X. (2015). Inhibitory effects of kaempferol on the invasion of human breast carcinoma cells by downregulating the expression and activity of matrix metalloproteinase-9. Biochem. Cell Biol..

[187] Lee C.F., Yang J.S., Tsai F.J., Chiang N.N., Lu C.C., Huang Y.S., Chen C., Chen F.A. (2016). Kaempferol induces ATM/p53-mediated death receptor and mitochondrial apoptosis in human umbilical vein endothelial cells. Int. J. Oncol..

[188] Braicu C., Catana C., Calin G.A., Berindan-Neagoe I. (2014). NCRNA combined therapy as future treatment option for cancer. Curr. Pharm. Des..

[189] Gibb E.A., Brown C.J., Lam W.L. (2011). The functional role of long non-coding RNA in human carcinomas. Mol. Cancer.

[190] Prensner J.R., Chinnaiyan A.M. (2011). The emergence of lncRNAs in cancer biology. Cancer Discov..

[191] Liu G., Zheng X., Xu Y., Lu J., Chen J., Huang X. (2015). Long non-coding RNAs expression profile in HepG2 cells reveals the potential role of long non-coding RNAs in the cholesterol metabolism. Chin. Med. J..

[192] Jiang P., Wu X., Wang X., Huang W., Feng Q. (2016). NEAT1 upregulates EGCG-induced CTR1 to enhance cisplatin sensitivity in lung cancer cells. Oncotarget.

[193] Chen J., Hou R., Zhang X., Ye Y., Wang Y., Tian J. (2014). Calycosin suppresses breast cancer cell growth via ERβ-dependent regulation of IGF-1R, p38 MAPK and PI3K/Akt pathways. PLoS ONE.

[194] Chen J., Lin C., Yong W., Ye Y., Huang Z. (2015). Calycosin and genistein induce apoptosis by inactivation of HOTAIR/p-Akt signaling pathway in human breast cancer mcf-7 cells. Cell. Physiol. Biochem..

[195] Gupta R.A., Shah N., Wang K.C., Kim J., Horlings H.M., Wong D.J., Tsai M.C., Hung T., Argani P., Rinn J.L. (2010). Long non-coding RNA HOTAIR reprograms chromatin state to promote cancer metastasis. Nature.

[196] Prensner J.R., Iyer M.K., Balbin O.A., Dhanasekaran S.M., Cao Q., Brenner J.C., Laxman B., Asangani I.A., Grasso C.S., Kominsky H.D. (2011). Transcriptome sequencing across a prostate cancer cohort identifies PCAT-1, an unannotated lincRNA implicated in disease progression. Nat. Biotechnol..

[197] Ciruelos Gil E.M. (2014). Targeting the PI3K/AKT/mTOR pathway in estrogen receptor-positive breast cancer. Cancer Treat. Rev..

[198] (2000). Cancer multidrug resistance. Nat. Biotechnol..

[199] Wu C.P., Ohnuma S., Ambudkar S.V. (2011). Discovering natural product modulators to overcome multidrug resistance in cancer chemotherapy. Curr. Pharm. Biotechnol..

[200] Rodrigues T., Reker D., Schneider P., Schneider G. (2016). Counting on natural products for drug design. Nat. Chem..

[201] Limtrakul P., Chearwae W., Shukla S., Phisalphong C., Ambudkar S.V. (2007). Modulation of function of three ABC drug transporters, P-glycoprotein (ABCB1), mitoxantrone resistance protein (ABCG2) and multidrug resistance protein 1 (ABCC1) by tetrahydrocurcumin, a major metabolite of curcumin. Mol. Cell. Biochem..

[202] Chearwae W., Wu C.P., Chu H.Y., Lee T.R., Ambudkar S.V., Limtrakul P. (2006). Curcuminoids purified from turmeric powder modulate the function of human multidrug resistance protein 1 (ABCC1). Cancer Chemother. Pharmacol..

[203] Chearwae W., Anuchapreeda S., Nandigama K., Ambudkar S.V., Limtrakul P. (2004). Biochemical mechanism of modulation of human P-glycoprotein (ABCB1) by curcumin I, II, and III purified from Turmeric powder. Biochem. Pharmacol..

[204] Boumendjel A., di Pietro A., Dumontet C., Barron D. (2002). Recent advances in the discovery of flavonoids and analogs with high-affinity binding to P-glycoprotein responsible for cancer cell multidrug resistance. Med. Res. Rev..

[205] Conseil G., Baubichon-Cortay H., Dayan G., Jault J.M., Barron D., di Pietro A. (1998). Flavonoids: A class of modulators with bifunctional interactions at vicinal ATP- and steroid-binding sites on mouse P-glycoprotein. Proc. Natl. Acad. Sci. USA.

[206] Zhou S., Lim L.Y., Chowbay B. (2004). Herbal modulation of P-glycoprotein. Drug Metab. Rev..

